# Low Serum Pyridoxine Levels Worsen Seizure Control in Adult Epilepsy Patients

**DOI:** 10.7759/cureus.25669

**Published:** 2022-06-05

**Authors:** Schweta Rane, Sama Elrahi, Joseph Villarreal, Haneef Zulfi, Xiang Fang, Daniel Graf, Rafael Rodriguez, Amanda Garza, Neeharika Thottempudi, Prashant Rai, Todd Masel

**Affiliations:** 1 Neurology, University of Texas Medical Branch, Galveston, USA; 2 Neurology, Baylor College of Medicine, Houston, USA; 3 Neurology, Geisinger Commonwealth School of Medicine, Scranton, USA; 4 Neurology, St. Joseph's Hospital, Tampa, USA

**Keywords:** vitamin b6, breakthrough seizures, poorly controlled epilepsy, vitamin deficiency, pyridoxine

## Abstract

Background: Vitamin B6 (pyridoxine) is an important cofactor in the process by which glutamic acid decarboxylase (GAD) converts the excitatory, pro-epileptogenic neurotransmitter, glutamate, into the inhibitory, anti-epileptogenic neurotransmitter, gamma-aminobutyric acid (GABA). This concept has been established in infants with pyridoxine-dependent epilepsy as well as adult patients with other epilepsy subtypes who presented with medication-resistant status epilepticus, with both patient groups experiencing cessation of seizure activity following pyridoxine administration. Given our knowledge of the role of vitamin B6 in the conversion of glutamate to GABA, its effect on seizure control in infants with specific epilepsy subtypes, reports of adult-onset seizures associated with vitamin B6 deficiency, and vitamin B6’s role in terminating status epilepticus in adult patients with other types of epilepsy, we suspect that low vitamin B6 levels in adult epilepsy patients may correlate with poor seizure control across all epilepsy subtypes. This study seeks to determine whether there is a relationship between pyridoxine levels and the level of seizure control in adults with epilepsy, regardless of their seizure type.

Methods: After obtaining institutional review board approval, we prospectively enrolled 32 patients (age range: 25-57 years) with epilepsy who presented to our clinic. Patients who did not meet the study criteria or who were diagnosed with psychogenic non-epileptic seizures (PNES) were excluded from the study (n = 2). Patients were classified as well-controlled (WC) or poorly controlled (PC) based on the absence or presence of a seizure within the last three months, respectively. After classification as WC or PC, pyridoxine serum levels and anti-seizure medication (ASM) levels were drawn in that clinic visit, following patient consent. All patients were contacted regarding pyridoxine and serum ASM levels, and patients that were found to be deficient in pyridoxine were treated with appropriate supplementation. At the end of the recruitment period, we performed analyses to determine if there was a statistically significant relationship between PC status and serum pyridoxine levels.

Results: Of 32 patients, two patients were diagnosed with psychogenic non-epileptic events and were subsequently excluded. Of 30 patients, 10 had PC epilepsy. Median (interquartile range) serum B6 levels were 35.8 (26.8-54.2) in patients with WC epilepsy and 17.5 (10.1-41.3) in patients with PC epilepsy (P = 0.11). In the PC group, 6/10 (60%) of the patients demonstrated low serum pyridoxine compared to 3/20 (15%) in the WC group (P = 0.03).

Conclusion: There was a statistically significant relationship between serum pyridoxine levels and seizure control. If appropriate, pyridoxine supplementation should be considered, especially in critically ill adult patients with refractory or PC seizures despite good adherence to ASMs.

## Introduction

Vitamin B6 (pyridoxine) is an important cofactor in the process by which glutamic acid decarboxylase (GAD) converts the excitatory, pro-epileptogenic neurotransmitter, glutamate, into the inhibitory, anti-epileptogenic neurotransmitter, gamma-aminobutyric acid (GABA) [1]. This concept has been established in infants with pyridoxine-dependent epilepsy in addition to other causes of infantile spasms, with vitamin B6 administration resulting in the cessation of seizures [2,3]. Additionally, reports of adult patients with epilepsy subtypes other than pyridoxine-dependent epilepsy who presented with anti-seizure medication-resistant status epilepticus responded to pyridoxine administration with subsequent termination of seizures [4]. Given our knowledge of the role of vitamin B6 in the conversion of glutamate to GABA, its effect on seizure control in infants with specific epilepsy subtypes [2,3], reports of adult-onset seizures associated with vitamin B6 deficiency [1,5,6], and vitamin B6’s role in terminating status epilepticus in adult patients with other types of epilepsy [4,5], we suspect that low vitamin B6 levels in adult epilepsy patients may correlate with poor seizure control across all epilepsy subtypes. This study seeks to determine whether there is a relationship between pyridoxine levels and the level of seizure control in adults with epilepsy, regardless of their seizure type.

## Materials and methods

Approval for this study was obtained from the University of Texas Medical Branch Institutional Review Board (IRB#16-0221). We enrolled patients with epilepsy, regardless of seizure subtype, who presented consecutively to our clinic and consented to participate in the study. Pregnant women were excluded from the study. Patients were classified into two groups, namely, well-controlled (WC) and poorly controlled (PC), based on the absence or presence of an unprovoked seizure episode within the last three months, respectively. Adherence to the anti-seizure medications (ASMs) was self-reported and was classified as good if the patient reported no instances of a missed dose. Patients who had breakthrough seizures due to extenuating circumstances, such as a missed dose of medication, severe sleep deprivation, or systemic illness, were excluded. Collected data included the type of epilepsy, seizure type, presence of seizure in the last three months, concurrent illness, medication compliance, whether ASM levels were in the therapeutic range, serum pyridoxine levels, EEG findings available during study enrollment, and MRI and CT head findings.

After classification into WC and PC groups, the patients had serum pyridoxine and ASM levels drawn on the same day. Nearly every patient had therapeutic ASM levels except one patient with unknown values. Low pyridoxine levels were defined as less than 20 nmol/L. Patients who were found to have a pyridoxine deficiency were treated with appropriate supplementation [7] regardless of the level of seizure control, and all patients received appropriate ASM therapy commensurate to the condition.

Fisher’s exact test was used to analyze the relationship between low pyridoxine levels and PC epilepsy, the relationship between patient's sex and seizure control, the median number of ASMs in each group, and to determine the statistical significance of the findings. The Wilcoxon rank-sum test was used to compare the median age in the two groups and the median number of ASMs in each group. All analyses were performed using IBM SPSS Statistics version 28.0.1.1 (IBM Corp., Armonk, NY).

## Results

Of the 32 patients that were initially enrolled and consented, two patients were excluded as they did not meet the study criteria, having been found to have psychogenic non-epileptic events. Out of 30 patients meeting the study criteria, 10 were determined to have PC epilepsy; six of these patients had low pyridoxine levels. The prevalence of low serum pyridoxine levels in the PC group starkly contrasted with that of the WC group, with only 15% of the WC population displaying a pyridoxine deficiency. Additionally, the majority (6/9) of patients with low serum pyridoxine levels had been classified as having PC epilepsy.

Statistical analysis using the Fisher’s exact test found that there was a statistically significant relationship between low pyridoxine levels and PC epilepsy (P = 0.03). However, there was no statistically significant correlation between sex and seizure control (P = 0.44). The Wilcoxon rank-sum test was used to compare the median age and number of ASMs between the two seizure groups (WC and PC), and no significant association was found between age and seizure control (P = 0.98) or the number of ASMs and seizure control (P = 0.25). Table 1 summarizes the relationship of various patient demographic factors with the degree of seizure control.

**Table 1:** Demographic characteristics and statistical analyses * Statistically significant relationship between low pyridoxine levels and PC epilepsy. IQR: interquartile range; ASM: anti-seizure medication.

	Poorly controlled epilepsy	Well-controlled epilepsy	Significance (p-value)
Serum pyridoxine level, median (IQR)	17.5 (9.9-41.7)	35.8 (26.5-56.1)	0.11
Low pyridoxine	6 (60%)	3 (15%)	0.03*
High and normal pyridoxine	4 (40%)	17 (85%)	0.35
High	1 (10%)	1 (5%)	
Normal	3 (30%)	16 (80%)	
Age (median IQR)	34.0 (24.8-51.0)	31.5 (25.0-56.5)	0.98
Sex			0.44
Female	6 (60%)	8 (40%)	
Male	4 (40%)	12 (60%)	
Number of ASMs, median (IQR)	3 (2.0-3.0)	2 (1.0-3.0)	0.25

## Discussion

We conducted a single-center, cross-sectional study to measure the relationship of serum pyridoxine levels with the level of seizure control in adult patients with histories of PC epilepsy. The results of this study indicate a statistically significant relationship between low serum pyridoxine levels and PC seizures despite patient-reported adherence and access to prescribed ASMs.

Pyridoxine's mechanism of action

Pyridoxine may influence seizure activity through a variety of mechanisms. Lower serum pyridoxine may imply lower activity of GAD, thus resulting in increased glutamate and lower GABA in the central nervous system (CNS). Pyridoxine is an important cofactor in the process by which GAD converts the excitatory, pro-epileptogenic neurotransmitter, glutamate, into the inhibitory, anti-epileptogenic neurotransmitter, GABA [1]. Seizures and epilepsy are associated with low levels of GABA as low GABA results in decreased levels of inhibition in the cerebral cortex [8]. As such, low levels of GABA lead to cell depolarization, thus increasing seizure activity. GABA’s effect is further evidenced by the fact that medications with GABA agonist activity, such as phenobarbital and valproic acid, are used for the treatment of seizures, and an abrupt withdrawal from medications, such as benzodiazepines, which are GABA type A (GABAA)-positive allosteric modulators, can provoke seizures [7,9]. Interestingly, increases in glutamate and decreases in GABA concentrations have been observed in the aged brain. Despite fluctuations in the levels of these neurotransmitters, invasive studies in rats have suggested that pyridoxine may also restore the activity of cerebral GAD to levels found in young animals, possibly preventing glutamate neurotoxicity during aging [10].

Evidence of pyridoxine deficiency implicated in seizures is plentiful in the literature. Previous studies, especially in the pediatric population, have documented pyridoxine-dependent epilepsy in which pyridoxine administration was successfully used for seizure cessation [2,3,11]. Among adults, case reports have suggested increased pyridoxine requirements, likely resulting from dietary deficiency, liver disease, pregnancy, and certain medications. After successfully treating pharmaco-refractory seizures with pyridoxine, these articles concluded that pyridoxine deficiency can be a standalone etiology for epilepsy [1]. Similarly, a case report by Morrow et al. demonstrated status epilepticus in a 25-year-old patient caused by isoniazid toxicity, which had led to pyridoxine deficiency [12]. Gerlach et al. also described three such patients as becoming seizure-free within 24-48 hours of intravenous pyridoxine followed by oral pyridoxine administration [13].

Limitations

The current study had several limitations. First, the study had a relatively small sample size. As the population size limited us to univariate statistics, it was difficult to monitor for confounding variables, such as the effect of ASM concentrations or drug interactions on the level of seizure control. Ideally, in a larger, prospective study, multivariate analyses would be conducted, enabling more robust conclusions to be drawn. Additionally, there was no scheduled patient follow-up after pyridoxine supplementation. This lack of follow-up may have hampered our ability to observe interval improvements in seizure control. Finally, the prescribed treatment regimen may also play a role in seizure control. In our study, the WC and PC patient groups did not have statistically significant differences in the median number of ASMs. However, with a larger population size, this could change.

## Conclusions

There was a statistically significant relationship between low serum pyridoxine levels and poor seizure control. Although pyridoxine deficiency-triggered seizures are rarely documented in adults, if appropriate, pyridoxine supplementation should be considered, especially in critically ill adult patients with refractory or PC seizures despite good adherence to ASMs.

## References

[1] Tong Y (2014). Seizures caused by pyridoxine (vitamin B6) deficiency in adults: a case report and literature review. Intractable Rare Dis Res.

[2] Hunt AD Jr, Stokes J Jr, McCrory WW, Stroud HH (1954). Pyridoxine dependency: report of a case of intractable convulsions in an infant controlled by pyridoxine. Pediatrics.

[3] Agadi S, Quach MM, Haneef Z (2013). Vitamin-responsive epileptic encephalopathies in children. Epilepsy Res Treat.

[4] Valle-Morales L, Cortés-Cros E, Santana A, Barber M, Figueras T, García-Hernández JA (2009). Epileptic status refractory to conventional treatment caused by vitamin B6 deficiency. J Perinatol.

[5] Dave HN, Eugene Ramsay R, Khan F, Sabharwal V, Irland M (2015). Pyridoxine deficiency in adult patients with status epilepticus. Epilepsy Behav.

[6] Lee DG, Lee Y, Shin H (2015). Seizures related to vitamin B6 deficiency in adults. J Epilepsy Res.

[7] Bernstein AL (1990). Vitamin B6 in clinical neurology. Ann N Y Acad Sci.

[8] Chapman AG (2000). Glutamate and epilepsy. J Nutr.

[9] Treiman DM (2001). GABAergic mechanisms in epilepsy. Epilepsia.

[10] Messripour M, Mesripour A (2011). Effects of vitamin B6 on age associated changes of rat brain glutamate decarboxylase activity. Afr J Pharm Pharmacol.

[11] Gospe SM Jr (2021). Pyridoxine-dependent epilepsy - ALDH7A1. GeneReviews®.

[12] Morrow LE, Wear RE, Schuller D, Malesker M (2006). Acute isoniazid toxicity and the need for adequate pyridoxine supplies. Pharmacotherapy.

[13] Gerlach AT, Thomas S, Stawicki SP, Whitmill ML, Steinberg SM, Cook CH (2011). Vitamin B6 deficiency: a potential cause of refractory seizures in adults. JPEN J Parenter Enteral Nutr.

